# Balancing Care and Chronic Illness: A Conceptual Framework for Older Caregivers of Advanced Illness Patients.

**DOI:** 10.1093/geroni/igaf122.3467

**Published:** 2025-12-31

**Authors:** Hyeyoung Park, Joohyun Chung

**Affiliations:** University of Massachusetts Amherst, Amherst, Massachusetts, United States; University of Massachusetts Amherst, Amherst, Massachusetts, United States

## Abstract

Aging caregivers are a growing yet under-recognized group. Many older caregivers provide care for individuals with advanced illness while also managing their own chronic conditions. Self-management plays a crucial role in chronic illness, and illness perception influences an individual’s self-management. However, the caregiving context—particularly when caring for someone with advanced illness—may alter how caregivers perceive their own illness, subsequently affecting their self-management. Previous research has primarily categorized individuals as either patients with chronic conditions or caregivers, rather than considering these roles simultaneously. To address this gap, this paper develop a conceptual framework that integrates the caregiving context as a moderating or mediating factor between illness perception and self-management, supported by preliminary findings. We conducted an exploratory study (N = 25) assessing illness perception, caregiving strain, anticipatory grief, and self-management among older caregivers of individuals with advanced illness. Descriptive statistics and non-parametric analyses were used to identify patterns. Given the small sample size, results were interpreted as preliminary insights informing theoretical development rather than definitive conclusions. Preliminary findings indicate that while illness perception among caregivers is heightened, this heightened awareness does not necessarily lead to improved or diminished self-management, pointing to the potential influence of other mediating factors. While prior research has suggested that caregiving stress may reduce illness perception due to self-neglect, our data reveal a different pattern, that caregiving stress seems to actually increase illness awareness.

